# When Concerned Family Members Intervene in Elder Family Financial Exploitation: Goals and Outcomes

**DOI:** 10.1093/geroni/igaa057.2144

**Published:** 2020-12-16

**Authors:** Marlene Stum

**Affiliations:** University of Minnesota, Twin Cities, Saint Paul, Minnesota, United States

## Abstract

The role and experience of non-abusing concerned family members (CFMs) in elder family financial exploitation (EFFE) is largely unexplored. This paper examines the experience of “trying to do the right thing,” focusing on what CFM’s were trying to accomplish (motivating goals), and resulting outcomes utilizing data from a qualitative study of 28 CFMs (primarily female adult children of an older victim, and siblings of primary perpetrator). Five common goals appear to be motivating CFM involvement, driven by a priority to ensuring the victim’s quality of life, as well as the desire to honor and respect the victim’s wishes, protect the victim’s financial well-being, preserve family relationships, and deal with the perpetrator(s). CFM help-seeking resulted in a wide range of outcomes, from making a difference by connecting victims to supportive services and interrupting the financial exploitation, to mixed results, and in other cases frustration with no desirable outcomes.

